# Mitochondria in the middle of inter- organellar cross talk: insights from yeast to humans

**DOI:** 10.3389/fcell.2026.1820168

**Published:** 2026-06-17

**Authors:** Nicoletta Guaragnella, Maria Antonietta Di Noia, Giampaolo Morciano, Angela Primavera

**Affiliations:** Department of Biosciences, Biotechnologies, and Environment, University of Bari Aldo Moro, Bari, Italy

**Keywords:** ERMES complex, inter-organellar cross talk, MAMS, mitochondria, retrograde signaling pathway

## Abstract

Mitochondria are multifaceted organelles acting as energy, metabolic and signaling hubs in the cells. Their role as sensors, integrators and transducers of intra and extracellular inputs can drive many processes of stress response. Mitochondrial signal integration can occur at different levels involving functional and physical interactions. An example of functional interaction is the mitochondria-cytosol-nucleus cross talk, named retrograde pathway, which is evolutionary conserved from yeast to humans and allows metabolic rewiring in the presence of. mitochondrial dysfunction. On the other hand, Physical Interactions, such as mitochondria associated membranes (MAMs), specialized contact sites between mitochondria and the endoplasmic reticulum (ER), play pivotal roles in calcium signaling and stress response. Calcium is tightly regulated at MAMs and functions both as stress sensor and mediator. Impaired mitochondrial function can lead to dysregulated calcium flux, particularly from the ER to mitochondria, triggering retrograde signaling pathways that alter nuclear gene expression and support cell adaptation. In yeast, the ER–mitochondria encounter structure (ERMES) complex exemplifies conserved mechanisms facilitating organelle tethering and metabolic cross talk. Dysfunctional mitochondria have significant repercussion on MAMs architecture, and viceversa, which impact on cell stress response particularly in, but not limited to, neurodegenerative disorders and cancer. In this review, we provide an overview of mitochondria-centered inter-organellar cross talk underpinning stress adaptation across eukaryotes, from yeast to humans. This comparative approach allows us to focus on key regulatory nodes, which are emerging as potential therapeutic targets. Components, such as calcium-dependent effectors, transcription factors, tethering proteins, or mitochondrial carriers could enable selective modulation of stress responses.

## Introduction

Beyond their primary role as cellular powerhouses, mitochondria are metabolic and signaling hubs sustaining cell homeostasis. Their mechanisms of communication with other cellular compartments are emerging as a relevant aspect in biology and biomedical sciences offering a new perspective for the comprehension of mechanistic clues guiding cellular stress response upon a variety of physio-pathological contexts. At this regard, the Mitochondrial Information Processing System (MIPS), constituted by mitochondria together with the other cellular compartments, can sense and integrate endogenous and environmental inputs and transduce the signals into adaptive responses (Picard and Shirihai, 2022). Mitochondrial signal integration occurs at multiple levels, involving both functional and physical interactions among cellular compartments, including molecular trafficking and membrane contact sites (MCS). MCS between mitochondria and other compartments have been well described in different species and compelling evidences suggest that MCS structural and functional defects may play a role in the pathogenesis of many human diseases. The mito-nuclear communication occurs in two directions, from the nucleus to the mitochondria, anterograde, and from the mitochondria to the nucleus, retrograde, and coordinates transcriptional responses that modulate the expression of specific genes required for mitochondrial biogenesis/activity and cell adaptation, respectively (Quirós et al., 2016). The peroxisome-mitochondria connection includes the metabolic cooperation in fatty acids metabolism, which is conserved in mammals and fungi. In addition, peroxisomes share a redox relationship with mitochondria contributing to cellular reactive oxygen species (ROS) homeostasis and affecting cell fate (Shai et al., 2018). However, the molecular architecture and functional specificity of mitochondria–peroxisome contact sites remain poorly defined. Communication between the ER and mitochondria is essential for the control of cellular metabolism and protein homeostasis. ER-MITO contacts regulate various cellular activities, such as iron homeostasis, innate immune response, metabolite exchange, and transfer of Ca2+ and lipids. In mammalian cells, the physical contact points between mitochondria and ER are called MAMs, mitochondria-associated membranes. Disruption of MAMs integrity has been associated to bioenergetic deficits, oxidative stress and apoptosis (Grimm, 2012). In lower eukaryotes, like yeast, ER-mitochondria connections occur through a complex named ERMES, required not only for lipid and metabolite transfer but also for proper segregation of the mitochondrial genome (Grimm, 2012; Murley et al., 2013). Mitochondria can interact with lysosomes or vacuoles through different processes, such as mitophagy, which is the selective intracellular degradation and recycling of damaged mitochondria, or ROS-mediated pathways leading to release of mitochondrial proteins and eventually cell death (Wong et al., 2019; Ungermann, 2015). In yeast, the contact sites between vacuole and mitochondria (vCLAMPs) are important for mitochondrial function, facilitating ions and metabolite exchange, especially phospholipids, and working in tandem with ERMES. Impairment of vCLAMPs leads to expansion of ERMES and viceversa. The absence of vCLAMPs leads to defects in respiration or reduced mitochondrial membrane potential. They are also implicated in metabolic stress responses, such as nutrient deficiency or mitophagy (Elbaz-Alon et al., 2014). Beyond ER and vacuole contacts, mitochondria interact with plasma membrane through mitochondria–ER–cortex anchors (MECA), which tether mitochondria to the cortical ER and the plasma membrane (Casler et al., 2025). These contacts contribute to mitochondrial positioning and have been shown to influence the number and spatial distribution of ERMES assemblies, highlighting the importance of cellular architecture and organelle positioning in the regulation of inter-organelle contact sites. Mitochondria-associated contact sites are further integrated into a broader inter-organelle network through shared regulatory factors. At this regard, nuclear–vacuolar junctions (NVJs) do not directly contact mitochondria but influence mitochondrial contact site organization in response to the cellular metabolic state.

In this review, we provide an overview of mitochondria as central hubs in inter-organelle cross talk, focusing on stress adaptation mechanisms across eukaryotes, from yeast to humans. In particular, we chose to shed light on mitochondrial retrograde signaling as an example of functional interaction and MAMs/ERMES for organelle tethering in different cellular stress contexts. Attention has been paid to molecular determinants that could represent potential therapeutic targets for selective modulation of stress response signaling pathways.

### Mitochondrial retrograde signaling and stress response from yeast to humans

**FIGURE 1:**
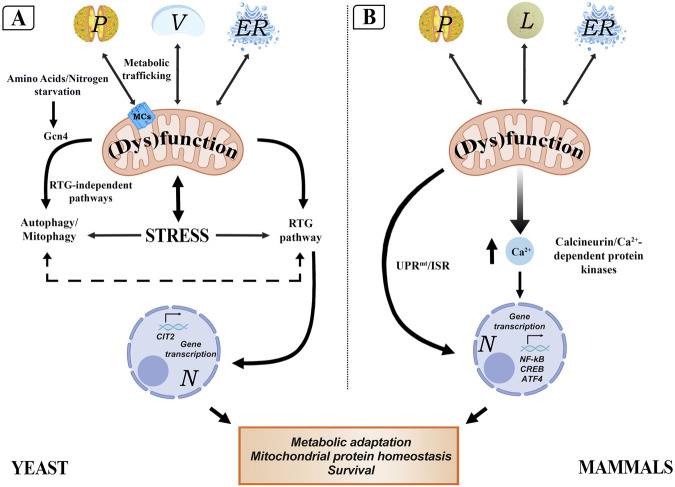
Functional interactions: retrograde signaling pathways in yeast and mammals. Schematic representation of mitochondria-centered retrograde signaling pathways in *Saccharomyces cerevisiae* and mammals under stress conditions. **(A)** Yeast: mitochondria act as a central hub integrating multiple intracellular inputs, including metabolic trafficking mediated by mitochondrial carriers (MCs) and communication with other organelles such as peroxisomes (P), vacuole (V), and endoplasmic reticulum (ER). Mitochondrial dysfunction activates the retrograde (RTG) pathway, driving nuclear (N) transcriptional reprogramming to support metabolic adaptation and survival. In parallel, amino acid or nitrogen starvation triggers an RTG-independent pathway mediated by Gcn4, promoting autophagy and mitophagy. The interplay between RTG-dependent and independent pathways (dashed line) remains to be elucidated. **(B)** Mammals: mitochondrial dysfunction triggers Ca^2+^-dependent signaling pathways involving calmodulin and calcineurin, which regulate nuclear gene transcription through transcription factors including NF-κB, CREB, and ATF4. Concurrently, mitochondrial stress activates the mitochondrial unfolded protein response (UPR^mt^) and the integrated stress response (ISR). Overall, these networks coordinate mitochondrial protein homeostasis, metabolic rewiring and stress adaptation, ultimately influencing cell fate.

A well-studied example of functional interaction is the mitochondria-cytosol-nucleus cross talk which regulates cell adaptation to mitochondrial dysfunction. The so-called retrograde pathway is evolutionarily conserved from yeast to humans and allows metabolic rewiring by activating the expression of specific genes upon mutations of mitochondrial DNA (mtDNA) or electron transport chain (ETC.) impairment (Butow and Avadhani, 2004; Lu et al., 2009; Quirós et al., 2016). Mitochondrial retrograde signaling has been discovered and characterized in its molecular details in the budding yeast *Saccharomyces cerevisiae*, which is an ideal model organism to study the impact of mitochondrial functionality on inter-organellar cross talk and cell stress response. In fact, it is a facultative anaerobe able to grow and survive in the presence of different mitochondrial dysfunctions, ranging from defects in ETC and/or OXPHOS, to partial or complete lack of mtDNA. Retrograde Genes (RTG), including *RTG1*, *RTG2* and *RTG3*, are involved in the metabolic reprogramming for adaptation to mitochondrial dysfunctions or to several stress conditions, including acid stress, ER stress, as well as oxidative and osmotic stress (Liu and Butow, 2006; Guaragnella et al., 2018; Guaragnella et al., 2019; Hijazi et al., 2020; Torelli et al., 2015; Lee et al., 2021). In particular, recent evidence in yeast, identifies citrate trafficking as a key regulatory node in RTG-dependent osmoadaptation, highlighting a functional axis between mitochondria and peroxisomes and underscoring the importance of mitochondrial carriers in stress adaptation (Di Noia et al., 2024; Primavera et al., 2025). The impact of metabolic compartmentalization and fluxes between mitochondria, cytoplasm and other organelles in the stress response of eukaryotes is still under-investigated, although it represents a key point for the modulation of cytoprotective strategies. At this regard, the proper functioning of metabolites and ions transport across the mitochondrial membranes is likely critical not only for maintaining homeostasis but also for coordinating inter-organelle communication under stress conditions. An RTG-independent retrograde signaling pathway related to Gcn4 and amino acid metabolism has also been described (Guaragnella and Butow, 2003). Beyond its role in amino acid metabolism, Gcn4 is a positive regulator of selected autophagic genes when TOR is inactive (Metur and Klionsky, 2024 and references therein), suggesting a potential intersection between retrograde signaling and autophagic processes. However, how retrograde pathways functionally integrate with autophagy and mitophagy in determining cell fate remains unclear. Interestingly, several lines of evidence highlight how intracellular ions levels, including manganese or iron, can affect recycling processes and retrograde signaling (Nicastro et al., 2022; Romero et al., 2021) (Figure 1 panel A). Therefore, intracellular ion homeostasis has emerged as an additional layer modulating both retrograde signaling and mitochondrial quality control.

Although mammalian orthologs of yeast *RTG* genes have not been found, many adaptive strategies to mitochondrial dysfunction are conserved, with some specific differences. Intracellular Ca^2+^ levels represent a major driver of the retrograde response and mediate the nuclear translocation of transcription factors, such as NF-κB, NFAT, CREB and HnRNP A2 through a calcineurin-mediated mechanism or the direct activation of Ca^2+^-dependent protein kinases, such as PKC, JNK, MAPK, and CAMKIV (Butow and Avadhani, 2004). In all cases, these pathways converge on the upregulation of genes affecting several cellular functions, including apoptosis and multi-drug resistance, invasion and Epithelial to Mesenchymal Transition (EMT) (Guerra et al., 2017). Specific studies have been focused on the correlation between tumorigenesis and mtDNA mutations/content, indicating that the latter might be determinant for the activation of oncogenic pathways at early stages of tumor progression (Kopinski et al., 2021). Another well studied example of retrograde signaling comes from the proteostatic cross talk among mitochondria, cytosol and nucleus. This is activated by the accumulation of abnormal proteins within the mitochondrial matrix and characterized by a conserved mechanism leading to the induction of chaperones and proteases (Charmpilas et al., 2025). In *Caenorhabditis elegans*, the mitochondrial Unfolded Protein Response (UPR^mt^) restores mitochondrial functionality through a mechanism of chromatin remodeling and transcriptional upregulation dependent on ATFS-1. In mammals, the UPR^mt^ maintains protein homeostasis in mitochondria to enhance mitochondrial function. Compelling evidences highlight that this stress pathway is coupled to Integrated Stress Response (ISR) which allow adaptive metabolic changes to improve mitochondrial functionality (Anderson and Haynes, 2020). Notably, ISR activates ATF4, the functional homologue of Gcn4, which is involved in stress recovery through metabolic adaptation or autophagy activation. This suggests an evolutionarily conserved mechanism of retrograde stress response in which mitochondria might activate a form of survival loop strictly dependent on the entity of the dysfunction, in terms of intensity and persistence. This will dictate the cell fate towards adaptation or cell death by involving different mechanisms, such as autophagy/mitophagy, homeostatic control of mitochondrial proteome, genetic and epigenetic responses, apoptosis. Moreover, the modulation of ISR and its downstream effectors has been shown to be effective in various disease models (Katow and Ryoo, 2024) (Figure 1 *panel B*).

Collectively, these findings position mitochondria as central regulators of a multilayered stress response network, in which the outcome depends on the nature and extent of mitochondrial dysfunction.

### Mitochondria-endoplasmic reticulum contact sites from ERMES to MAMs

**FIGURE 2:**
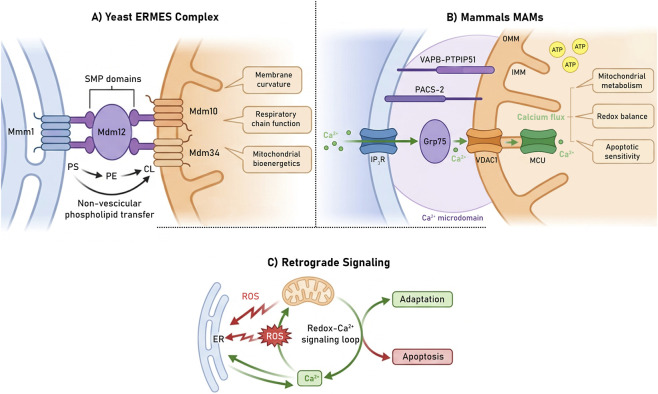
Physical interactions: mitochondria–ER contact sites and retrograde signaling. **(A)** Yeast ERMES. In *Saccharomyces cerevisiae*, ER–mitochondria contacts are mediated by the ERMES complex (Mmm1, Mdm12, Mdm10, Mdm34), which enables non-vesicular phospholipid transfer (PS, PE, CL) and supports mitochondrial structure and function. **(B)** Mammalian MAMs. In mammals, MAMs are maintained by multiple tethers (e.g., VAPB–PTPIP51, PACS-2) and mediate Ca^2+^ transfer from ER to mitochondria via the IP_3_R–Grp75–VDAC1– MCU axis, regulating metabolism and stress responses. **(C)** Retrograde signaling. Mitochondrial ROS and Ca^2+^ form a feedback loop at ER–mitochondria interfaces that promotes metabolic adaptation or, under sustained stress, triggers apoptosis.

Membrane contact sites (MCS) underpin mitochondrial communication by enabling direct exchange of metabolites and signals between organelles. Much of our current understanding of MCS biology originates from studies in *S. cerevisiae*, where key tethering complexes and regulatory factors have been identified. The best-characterized mitochondrial contact site is the ER–mitochondria encounter structure (ERMES), a yeast-specific multisubunit complex that bridges the ER membrane and the mitochondrial outer membrane. ERMES is composed of four core subunits that stabilize discrete ER–mitochondria contact sites: the ER membrane protein Mmm1, the cytosolic protein Mdm12, and the mitochondrial outer membrane-associated proteins Mdm10 and Mdm34 (Kornmann et al., 2009; Lang et al., 2015) (Figure 2, *panel A*). Synaptotagmin-like mitochondrial lipid-binding protein (SMP) domains are present in three of its subunits. SMP domains assemble into a lipid-binding scaffold capable of accommodating phospholipid species. This architecture supports a well-established role for ERMES in facilitating non-vesicular lipid exchange between the ER and mitochondria. (AhYoung et al., 2015; Kawano et al., 2018; Mavuduru et al., 2024). In fact, loss of ERMES results in altered mitochondrial phospholipid composition, including defects in phosphatidylserine (PS) import and downstream perturbations in phosphatidylethanolamine (PE) and cardiolipin (CL) homeostasis which are critical for membrane curvature, respiratory supercomplex stability and mitochondrial bioenergetic efficiency. Therefore, these lipid imbalances are accompanied by severe mitochondrial morphological defects and impaired respiratory growth.

ERMES function is also modulated by regulatory factors that control its organization and dynamics, including the mitochondrial GTPase Gem1, a homolog of mammalian Miro proteins. Under conditions of ER stress or mitochondrial dysfunction, ERMES clusters undergo active remodeling characterized by increased dispersion and enhanced lateral mobility along the mitochondrial surface, without complete loss of ER–mitochondria contacts (Kornmann et al., 2011; Stroud et al., 2011). Functionally, ERMES remodeling correlates with reduced ER-to-mitochondria lipid flux, decreased mitochondrial PE and CL levels, and impaired respiratory performance, suggesting that contact site plasticity represents an adaptive mechanism to tune inter-organelle communication under stress conditions (Kakimoto-Takeda et al., 2022).

The assembly of ERMES in discrete punctate foci has suggested its involvement in mitochondrial biogenesis, genome maintenance and organelle inheritance. Accordingly, its disruption causes abnormal mitochondrial morphology, respiratory defects, impaired protein import, and mtDNA instability. ERMES has also been implicated in mitochondrial dynamics, mitophagy and stress-induced mitochondrial quality control, underscoring its function as a multifunctional hub integrating membrane contact formation with mitochondrial physiology (Murley et al., 2013; Böckler and Westermann, 2014; Smethurst and Cooper, 2017). However, it remains unclear whether these functions are directly regulated by ERMES or arise indirectly as a consequence of mitochondrial dysfunction. Interestingly, a link between ERMES and RTG-dependent retrograde signaling has been reported (Cirigliano et al., 2019); in respiration-deficient yeast cells, including rho° cells lacking mtDNA or cells treated with respiratory chain inhibitors, activation of the RTG pathway induces transcriptional upregulation of ERG27, a 3-keto sterol reductase in the ergosterol biosynthetic pathway. Under these conditions, ERG27 redistributes from lipid droplets to the ER membrane, reflecting a spatial rewiring of sterol biosynthesis in response to mitochondrial stress. This rewiring correlates with reduced ergosterol levels in respiration-deficient cells compared with wild-type controls. Interestingly, in ERMES mutants, particularly cells lacking the subunit Mdm34, reduced ergosterol levels appear to result primarily from mitochondrial dysfunction and loss of mtDNA rather than a direct defect in ERMES-mediated sterol transport. These findings support a framework in which ERMES integrity maintains mitochondrial function, which in turn modulates sterol homeostasis via retrograde signaling. This model highlights how mitochondrial status can indirectly shape lipid metabolism, linking organelle function, inter-organelle communication, and metabolic adaptation.

ERMES may also influence other metabolism-linked stress signals, including iron and heme homeostasis. ERMES dysfunction triggers the Aft1/Aft2 iron-deficiency response, phenocopying defects in Fe–S cluster assembly and highlighting a potential route for mitochondria-to-ER communication independent of Ca^2+^ signaling (Xue et al., 2017) Mitochondrial inner membrane carriers, such as Rim2p, contribute indirectly to mitochondrial cofactor assembly and may integrate with ERMES-mediated regulation of mitochondrial function, further supporting the concept that ER–mitochondria contact sites act as hubs integrating structural and metabolic inputs (Marobbio et al., 2006).

MAMs are stable yet flexible tethering interfaces between ER and mitochondria maintained by protein complexes and lipid-transfer systems. (Figure 2, *panel B*). Unlike ERMES, MAMs are not defined by a single conserved complex but rather by a dynamic ensemble of proteins, reflecting a higher level of structural and functional complexity in mammalian cells. They are crucial hubs for inter-organelle communication in mammalian cells, allowing exchange of signals and metabolites. MAMs serve as platforms that regulate a wide range of cellular processes (Marchi et al., 2018; Morciano et al., 2018). One of the most prominent roles is in Ca^2+^ signaling (Rizzuto et al., 1998); this localized Ca^2+^ transfer is essential for mitochondrial bioenergetics. In addition to their roles in metabolism, MAMs are now recognized as critical regulators of cellular stress responses, autophagy and apoptosis (Lv et al., 2025; Tao et al., 2025; Landry et al., 2025).

The importance of MAMs extends to human health and disease, as disruptions in ER–mitochondria communication have been implicated in a variety of pathological conditions, such as neurodegenerative diseases, metabolic disorders, cardiovascular diseases and cancer (Marchi et al., 2018; Giorgi et al., 2018; Agyapong et al., 2024). However, the extent to which MAMs dysfunction plays a causal role in these conditions, rather than representing a secondary consequence of cellular stress, remains to be fully established. Taken together, these observations indicate that ER–mitochondria contact sites are not merely structural entities but dynamic regulatory hubs that integrate metabolic state and stress signaling. Their functional plasticity enables mitochondria to coordinate adaptive responses across cellular compartments, reinforcing their role as central orchestrators of inter-organelle communication.

### Calcium regulation at MAMs

Calcium signaling at MAMs exemplifies the broader principle of retrograde signaling allowed by the physical architecture of ER - mitochondria contacts. Rather than acting as passive diffusion sites, MAMs generate highly localized Ca^2+^ microdomains that couple organelle proximity to metabolic output and cellular responses (Marchi et al., 2020). At the mechanistic level, Ca^2+^ transfer is mediated by a core module composed of IP3Rs at the ER (Woll and Van Petegem, 2022), voltage-dependent anion channel 1 (VDAC) at the outer mitochondrial membrane (Hajnóczky et al., 2002) and the mitochondrial Ca^2+^ uniporter (MCU) at the inner membrane (De Stefani et al., 2011). However, the efficiency of this pathway does not simply depend on channel activity, but critically on the spatial organization imposed by tethering complexes, such as the glucose-regulated protein 75 (Grp75) bridge linking IP3Rs to VDAC1 (Szabadkai et al., 2006) (Figure 2 *panel* B).

This highlights a key concept: Ca^2+^ signaling at MAMs is a structural–functional unit, where modulation of tethering directly impacts signal propagation. For instance, proteins such as vesicle-associated membrane protein-associated protein B (VAPB) and protein tyrosine phosphatase interacting protein 51 (PTPIP51) (Ando et al., 2016; Benjamin Bonneau, 2016; De vos et al., 2012; Shiiba et al., 2025) or phosphofurin acidic cluster sorting protein 2 (PACS-2) (Nhung et al., 2023) not only stabilize contacts but dynamically tune Ca^2+^ flux by altering inter-organelle distance. Importantly, the field still lacks a clear hierarchy among these regulators. While numerous modulators (e.g., Sig-1R, GSK3β, FUNDC1) have been identified, their relative contribution to Ca^2+^ flux under physiological versus stress conditions remains poorly defined, raising the possibility that many act in a context-dependent rather than universal manner. Functionally, Ca^2+^ transfer at MAMs integrates at least three processes: mitochondrial metabolism, redox balance and apoptotic sensitivity. Disruption of mitochondrial function feeds back on this system at multiple levels, reducing ATP-dependent Ca^2+^ reuptake into the ER, impairing mitochondrial buffering capacity and altering contact dynamics. This positions Ca^2+^ not as an isolated pathway, but as a central integrator of structural and metabolic communication between the ER and mitochondria.

### Mitochondria - To - Endoplasmic reticulum retrograde signaling

Although mitochondrial retrograde signaling is universally recognized as the mechanism by which mitochondria communicate their functional status to the nucleus, recent studies have highlighted the ER as a key intermediate through which mitochondria signal dysfunction. This happens in mammals through three tightly coupled layers: 1) the above-mentioned Ca^2+^ fluxes, (2) dynamic remodeling of physical contacts, and (3) redox signals. Rather than acting as independent pathways, these components form a coordinated signaling module that determines whether cells adapt or undergo apoptosis.

A central mechanism underlying this communication is the coupling between mitochondrial ROS production and ER Ca^2+^ release. Under physiological conditions, mitochondria undergo transient depolarization events which involve brief openings of the permeability transition pore. These events generate localized oxidative signals at ER–mitochondria interfaces. These microdomain-restricted ROS signals sensitize nearby IP3Rs, enhancing Ca^2+^ release from the ER and promoting rapid mitochondrial uptake (Booth et al., 2021). This establishes a redox–Ca^2+^ feedback loop, in which mitochondrial activity directly modulates ER signaling and, in turn, reinforces mitochondrial Ca^2+^ loading and metabolic output. Importantly, under stress conditions this feedback loop becomes amplified. Sustained mitochondrial damage increases both the frequency and duration of depolarization events, leading to prolonged oxidative bursts and excessive Ca^2+^ transfer. Although initially adaptive, this transition can shift the system toward apoptosis, promoting Bax activation, mitochondrial permeabilization and global bioenergetic collapse (Booth et al., 2021). However, the extent to which this mechanism operates *in vivo*, and whether it represents a universal or context-specific response, remains to be fully established. Notably, studies in *S. cerevisiae* indicate that cytosolic Ca^2+^ elevations are closely linked to ROS production, influencing mitochondrial permeability and programmed cell death (Figure 2 *panel C*). This suggests that the functional coupling between calcium flux and redox state at organelle interfaces may represent a conserved stress-response principle across eukaryotes, from yeast to mammals, despite differences in the underlying molecular components (Carraro and Bernardi, 2016).

Beyond signal propagation, mitochondrial stress also actively reshapes the physical architecture of ER–mitochondria contacts, thereby modulating signaling efficiency. For instance, phosphorylation of the tethering protein PTPIP51 enhances its interaction with VAPB, strengthening inter-organelle coupling under oxidative stress conditions (Shiiba et al., 2025). This structural reinforcement facilitates the transfer of lipid-derived radicals from mitochondria to the ER, reducing mitochondrial oxidative burden. These observations support the idea that contact site remodeling is not merely a consequence of stress, but an active component of retrograde signaling, tuning both the intensity and the nature of inter-organelle communication. In parallel, mitochondrial dysfunction engages canonical ER stress pathways, further illustrating the integration of functional and structural signals. ROS production and impaired respiration can activate ER stress sensors through signaling nodes such as GSK-3β, leading to induction of BiP, PERK, ATF4 and CHOP (Han et al., 2014). Similarly, the mitochondrial ubiquitin ligase MITOL (March5), when brought into proximity of the ER, modulates the activity of the unfolded protein response sensor IRE1α. By promoting K63-linked ubiquitination, MITOL restricts IRE1α oligomerization and biases signaling toward adaptive rather than pro-apoptotic outputs (Takeda and Yanagi, 2019). These findings highlight a key principle: mitochondrial status influences not only the activation but also the qualitative outcome of ER stress responses.

A further layer of complexity is added by the mitochondrial unfolded protein response (UPR^mt^), which coordinates transcriptional adaptation to proteotoxic stress. Mild mitochondrial dysfunction due to mtROS or the accumulation of protein precursors triggers increased ER–mitochondria coupling, enhancing Ca^2+^ transfer and boosting mitochondrial respiration. This suggests that structural tightening of MAMs represents an early adaptive response, supporting the energetic and proteostatic demands of stress recovery. However, whether increased coupling is universally protective or can become maladaptive under chronic stress conditions remains an open question. (Sutandy et al., 2023; Lopez- Crisosto et al., 2021).

Beyond these mechanisms, an additional layer of regulation emerges at the level of metabolic pathways. In mammalian cells, mitochondrial dysfunction has been shown to suppress the mevalonate pathway through altered ER sterol sensing, impairing SREBP2 activity and promoting degradation of key biosynthetic enzymes. Notably, these effects occur despite relatively stable total cholesterol levels, suggesting that organelle communication, rather than bulk metabolite abundance, governs metabolic regulation (Wall et al., 2022). A similar principle emerges in *S. cerevisiae*, where respiratory defects trigger ER redistribution of ERG27 via the RTG pathway, highlighting that organelle communication, rather than metabolite abundance alone, governs lipid metabolic regulation. This provides a mechanistic link between mitochondrial defects, ER signaling, and broader metabolic rewiring observed in aging and disease.

Taken together, these observations support a unified model in which mitochondria-to-ER retrograde signaling is not mediated by isolated pathways, but by an integrated network where redox signals, Ca^2+^ dynamics and contact site plasticity converge to regulate cellular fate. A major challenge for the field is to define the hierarchy and context-dependency of these mechanisms, as well as to determine how their dysregulation contributes to human disease.

### Targeting MAMs: Opportunities and challenges for therapeutic intervention

Insights from mammalian systems and *S. cerevisiae* converge on conserved mechanisms regulating ER–mitochondria communication, providing a conceptual framework for the identification of potential therapeutic targets. Although direct small-molecule modulation of ER–mitochondria contact sites has not yet been established in yeast due to structural differences with mammalian MAMs, yeast-based assays have been successfully used to identify compounds that rescue mitochondrial dysfunction in models of human mitochondrial disease, validating conserved pathways and guiding the selection of pharmacological targets in higher eukaryotes (Di Punzio et al., 2021). From a translational perspective, MAMs components represent attractive but challenging therapeutic targets. Experimental strategies have begun to emerge, although they remain largely confined to preclinical models. These approaches can be broadly categorized into three mechanistic classes: modulation of physical tethering, regulation of Ca^2+^ transfer and targeting of upstream signaling pathways that coordinately control both structural and functional aspects of MAMs.

Among tethering complexes, the VAPB–PTPIP51 axis is one of the most extensively studied, particularly in neurodegenerative diseases such as amyotrophic lateral sclerosis and frontotemporal dementia, where its disruption impairs ER–mitochondria coupling and Ca^2+^ homeostasis. Genetic restoration of this interaction rescues organelle contacts and normalizes multiple downstream pathways (De vos et al., 2012). To overcome the limitations of genetic approaches, small molecules such as LDC-3 have been developed to enhance PTPIP51 phosphorylation and reorganize MAM-associated complexes, modulating Ca^2+^ flux and metabolic signaling, with potential anti-proliferative or pro-apoptotic effects depending on cellular context (Dietel et al., 2019). Mitofusins (MFN1/2) represent another tethering target. Small-molecule MFN2 agonists or HR1-derived peptides can stabilize productive ER–mitochondria tethering, improving Ca^2+^ transfer, mitochondrial bioenergetics, and cellular homeostasis in neurodegenerative or metabolic disease models (Rocha et al., 2018). Conversely, MFN2-mimetic peptides can promote pro-apoptotic MAM configurations, enhancing mitochondrial Ca^2+^ uptake, a strategy of potential interest in cancer therapy (Zacharioudakis et al., 2022).

Several studies have targeted the Ca^2+^ transfer machinery directly. Pharmacological inhibition of MCU (e.g., Ru360, DS16570511) prevents mitochondrial Ca^2+^ overload and reduces apoptosis in ischemia–reperfusion and neurodegeneration models (Agyapong et al., 2024). However, these approaches lack spatial specificity, affecting global Ca^2+^ handling rather than MAM-restricted signaling, thus increasing off-target effects and limiting translational potential. An alternative strategy focuses on regulators of MAM integrity. GSK3β modulates ER–mitochondria coupling via VAPB–PTPIP51 interactions, and its inhibition restores contact sites and mitigates pathology (Gomez-Suaga et al., 2017). Small GTPases such as RHOA also regulate contact dynamics, suggesting additional therapeutic targets, although these pathways remain less defined.

A key limitation is the dual role of ER–mitochondria contacts: both excessive and reduced coupling are detrimental, affecting Ca^2+^ balance, bioenergetics, and cell survival. Therefore, the main challenge is to selectively tune MAM function in a context-dependent manner without disrupting overall cellular homeostasis.

## Conclusions and perspectives

While therapeutic strategies targeting MAMs are still in early stages, studies in yeast and mammalian systems reveal a conserved principle: mitochondria continuously communicate their functional state to other organelles through a combination of physical and functional contacts. In yeast, ERMES tethers mitochondria to the ER, coordinating lipid exchange, metabolic adaptation, and retrograde signaling via RTG-dependent and independent pathways. In mammalian cells, analogous structures, MAMs, mediate Ca^2+^ flux, lipid transfer, and redox signaling, which can feed back to the ER and trigger stress responses such as the UPR^mt^. Beyond calcium, other inter-organellar ion fluxes play key roles. Lactate can trigger ER magnesium release into mitochondria, (Daw et al., 2020); additionally, mitochondria are a major cellular iron reservoir, and disruption of ERMES can activate an iron starvation response (Xue et al., 2017). Despite differences in molecular components and pathway architecture, a unified conceptual framework emerges: mitochondrial health is continuously monitored and communicated through dynamic interactions, enabling cells to sense, adapt, and respond to mitochondrial stress. This conserved design underscores the central role of inter-organellar cross talk in maintaining cellular homeostasis and modulating stress response with respect to the intensity of the stress itself. Mitochondria- organelle contacts are implicated in many diseases, but their therapeutic potential remains under investigation. A key future challenge is the identification of molecular signatures, regulatory proteins, and metabolic determinants governing these interactions, in order to distinguish adaptive from maladaptive contact remodeling. Such knowledge could guide the development of targeted modulators capable of selectively enhancing or disrupting specific tethering events. Ultimately, a deeper understanding of inter-organelle cross talk will provide fundamental insights into cell biology and elucidate mechanisms underlying diverse human diseases (Table 1).

**TABLE 1:** Summary of major mitochondria-associated contact sites and functional interactions involved in cellular stress responses: key protein components, biological functions and their relevance to human disease from yeast to mammals.

Contact sites/ Pathways	Organisms	Key proteins/ Signaling nodes	Core functions	Role in stress Response	Disease relevance
MAMs (ER– mitochondria contact sites)	Mammals	VAPB–PTPIP51, MFN1/2, GRP75, IP3R–VDAC– MCU axis, GSK3β	ER–mitochondria tethering, Ca²⁺ transfer, lipid exchange	Integration of Ca²⁺ and redox signaling; regulation of metabolism vs. apoptosis	Neurodegeneration (ALS, Alzheimer’s), cancer, metabolic diseases
ERMES (ER– mitochondria encounter Structure)	Yeast	Mmm1, Mdm12, Mdm34, Mdm10, Gem1	Physical tethering, phospholipid transfer, mtDNA maintenance	Adaptive remodeling of contact sites; lipid homeostasis	Mitochondrial dysfunction, respiratory defects
Mito–nuclear Retrograde signaling	Yeast Mammals	RTG1/2/3, Gcn4 (yeast); NF-κB, NFAT, CREB, ATF4 (mammals)	Transcriptional regulation of nuclear genes	Metabolic rewiring	Cancer, aging, mitochondrial diseases
mtUPR/ISR axis	Mammals	ATF4, mitochondrial chaperones, ISR components	Proteostasis and stress recovery	Integration of mitochondrial dysfunction with transcriptional programme	Neurodegeneration, cancer
Ca²⁺ signaling module at MAMs	Mammals	IP3R, GRP75, VDAC1, MCU	ER-to-mitochondria Ca²⁺ flux	Control of bioenergetics vs. apoptotic switch	Ischemia, neurodegeneration
Redox signaling (ROS- mediated cross talk)	Yeast Mammals	ETC., complexes, ROS, redox-sensitive proteins	Signal transduction and feedback regulation	ROS–Ca²⁺ feedback loops; stress amplification or adaptation	Cancer, aging
